# COMPETENCY-BASED UNDERGRADUATE PALLIATIVE NURSING EDUCATION USING AN INTERPROFESSIONAL TEACHING APPROACH

**DOI:** 10.1093/geroni/igad104.2421

**Published:** 2023-12-21

**Authors:** Brandi Felderhoff, Catherine Mbango

**Affiliations:** Texas Woman’s University, Denton, Texas, United States; Texas Woman’s University, Houston, Texas, United States

## Abstract

The nursing workforce is expected to be “work ready” to address healthcare needs in diverse communities. Health inequities continue to persist, and access to healthcare services that promote well-being and improve quality of life is imperative. Nursing curricula that integrate competencies in palliative supportive care for patients with chronic complex illnesses are needed to improve health equity and promote global citizenship (AACN, 2021). The shift to competency-based learning is both an accreditation requirement, and essential to prepare nursing students for the NextGen NCLEX (Wolf, 2022). Through review of the AACN’s (2021) The Essentials: Core Competencies for Professional Nursing Education core competency of palliative and supportive care in the domain of population health for entry-level professional nurses, and framework from The Interprofessional Education Collaborative (IPEC) for team-based competencies to improve population health (IPEC, 2016), a nursing-social work education partnership is well-suited to address AACN’s Essentials utilizing IPEC’s four core competencies. This approach to teaching allows students to develop knowledge and competence in caring for patients with palliative or supportive care needs, effectively bridging the competency gap (Jones & West, 2017) with curricula developed through interprofessional collaboration (Gravina, 2017). Primary palliative nursing care is an interdisciplinary patient-centered approach that focuses on the management of physical, psychological, social, and spiritual care of patients and their families. The opportunity to learn through an interdisciplinary teaching approach better prepares students to examine their place within the team, and fully understand the related roles of their allied colleagues.

